# Evolutionary analysis of the *Moringa oleifera* genome reveals a recent burst of plastid to nucleus gene duplications

**DOI:** 10.1038/s41598-020-73937-w

**Published:** 2020-10-19

**Authors:** José Ojeda-López, Juan Pablo Marczuk-Rojas, Oliver Aleksandrei Polushkina, Darius Purucker, María Salinas, Lorenzo Carretero-Paulet

**Affiliations:** Department of Biology and Geology, University of Almería, Ctra, Sacramento s/n, 04120 Almería, Spain

**Keywords:** Computational biology and bioinformatics, Evolution, Genetics, Plant sciences

## Abstract

It is necessary to identify suitable alternative crops to ensure the nutritional demands of a growing global population. The genome of *Moringa oleifera*, a fast-growing drought-tolerant orphan crop with highly valuable agronomical, nutritional and pharmaceutical properties, has recently been reported. We model here gene family evolution in Moringa as compared with ten other flowering plant species. Despite the reduced number of genes in the compact Moringa genome, 101 gene families, grouping 957 genes, were found as significantly expanded. Expanded families were highly enriched for chloroplastidic and photosynthetic functions. Indeed, almost half of the genes belonging to Moringa expanded families grouped with their *Arabidopsis thaliana* plastid encoded orthologs. Microsynteny analysis together with modeling the distribution of synonymous substitutions rates, supported most plastid duplicated genes originated recently through a burst of simultaneous insertions of large regions of plastid DNA into the nuclear genome. These, together with abundant short insertions of plastid DNA, contributed to the occurrence of massive amounts of plastid DNA in the Moringa nuclear genome, representing 4.71%, the largest reported so far. Our study provides key genetic resources for future breeding programs and highlights the potential of plastid DNA to impact the structure and function of nuclear genes and genomes.

## Introduction

In order to ensure the food and energy supply of a growing world population, agricultural production must double by 2050. This is expected to be a huge challenge in the context of climate change, featured by unpredictable weather, including erratic precipitations and temperatures and alterations in CO_2_ levels. Furthermore, about 95% of the human caloric intake is based on only 30 crop species, of which wheat, maize, and rice provide the vast majority. Therefore, to diversify and stabilize the global food supply, enhance agricultural productivity, and tackle malnutrition must become a priority to achieve the United Nations’ 17 Sustainable Development Goals of decent lives for all on a healthy planet by 2030 (https://www.un.org/sustainabledevelopment/). At this respect, the African Orphan Crop Consortium (AOCC) emerged to promote the research and production of neglected or underutilized (orphan) local plants, but with great agronomic potential^1^. For this purpose, the consortium has selected 101 orphan species from indigenous crops of the African continent and other naturalized exotic species to sequence their genome and transcriptome, allowing the identification of genes of agronomic interest and associated molecular markers and ultimately, the development of plant improvement programs. The draft genomes of the first five selected orphan crops have been recently released, including that of the plant tree *Moringa oleifera, Vigna subterranea*, *Lablab purpureus*, *Faidherbia albida* and *Sclerocarya birrea*^2^.

The UN’s Food and Agriculture Organization (FAO), has promoted Moringa as a highly nutritious, fast growing and drought tolerant crop (https://www.fao.org/traditional-crops/moringa/en/). Originally from India, it is also an important crop in Ethiopia, Nigeria, Philippines and Sudan and its culture is expanding to a wide range of tropical and subtropical regions in Africa and the Americas^2–5^. Botanically, Moringa belongs to the order Brassicales, and together with other 13 species, conform the monotypic family Moringaceae. The leaves of Moringa provide a nutritious vegetable, with 20–30% protein content in the leaflets by dry weight^6^. Leaves, flowers and fruits are also rich in vitamins A, B and C and minerals, notably Ca and Fe. The seeds yield a high-oleic edible oil used in cooking, cosmetics, and as a machinery lubricant. After oil extraction, the remaining seed cake can be used to clarify turbid water or to increase protein in animal feed or crop fertilizer^4^. Used in traditional medicine since the ancient Egyptians, the plant produces a wide range of secondary metabolites, including carotenoids, alkaloids, chlorogenic acids, saponins, phenolics and flavonoids, for which diverse pharmacological roles as bioactive compounds are under study^4,7^. In particular, Moringa produces substantial amounts of glucosinolates, for which ongoing investigation is providing evidence of a wide range of medicinal properties, including antioxidant, anti-inflammatory, antibiotic, neuroprotective, cytoprotective, chemoprotective and cancer-suppressing^8–11^.

Comparative genome wide analysis of gene family size variation has become a common tool to get a first insight into the adaptive landscape of plant genomes, and has helped to identify gene and gene families at the origin of relevant biological adaptations and agronomical features in specific species^12–14^. Lineage-specific gene family expansions result from the acquisition of novel genes, which may evolve de novo from DNA sequences that were ancestrally non-genic, including transposable elements^15^, from events of horizontal gene transfer^16^, and most importantly, from different events of gene and genome duplication that are abundant in the plant lineage^17,18^.

Nuclear gene duplicates are the primary source of genetic material to evolve novel and/or specialized biological functions, including those that may be at the origin of adaptive traits. Most duplicate genes can be classified into two main groups according to the mechanism of origin. First, duplicates arising from whole genome duplications (WGDs), also known as polyploidizations, occur at specific times throughout evolution^19,20^, and result in the duplication of every gene in the genome. Second, duplicate genes originating from small-scale duplications (SSDs), notably tandem duplications, which are continuously occurring and only involve one to a few genes^17^. Most duplicated genes are expected to stochastically accumulate deleterious mutations, finally becoming a pseudogene or being eliminated from the genome (non-functionalization)^21^. Furthermore, WGD events are usually followed by rediploidization and fractionations, consisting of genomic rearrangements that result in genome downsizing and the loss and/or divergence of most duplicated genes, ultimately restoring the genome back to the original diploid state^22,23^. However, some gene duplicates can be retained in the genome for longer evolutionary times through the acquisition of novel or specialized functions^17,24^. This way, differential rates of gene duplication and subsequent retention or loss operating on specific plant lineages may contribute to significant expansion and contractions in specific gene families.

Because of the prominent role attributed to nuclear gene and genome duplications in providing the substrate for the evolution of novel or specialized gene and regulatory functions, the topic has devoted much attention^17,18,24^. In contrast, other potential genomic sources of evolutionary innovation and adaption are less studied. One such source is the well-known transfer of plastid (and other organelles) genes and DNA to the nuclear genome^25^. Using a classification of orthogroups in the Moringa genome and 10 other plant species representing the main flowering plant lineages, we modelled gene family turnover using the Maximum Likelihood (ML) framework implemented in Badirate^26^. Categorization of the functional space of expanded gene families revealed an outstanding enrichment in chloroplast-associated functions. A significant part of this expansion resulted from the repeated transfer of massive amounts of plastid DNA and plastid genes to the nuclear genome, rather than because of duplicates generated through SSD or WGD. We further discuss the potential role that plastid gene duplications may play in shaping the evolution of plant genome structure and function.

## Results

### Identification of Moringa-specific expanded and contracted gene families

We first obtained a classification of orthogroups in the Moringa genome and 10 plant species representing the main angiosperm plant lineages (Supplementary Table S1). These included the eudicots Arabidopsis and *Vitis vinifera* (grape), four eudicot AOCC crops whose genomes were reported together with that of Moringa (*F. albida*, *L. purpureus*, *S. birrea* and *V. subterránea*)^2^, the monocots *Oryza sativa* (rice) and *Zea mays* (maize), the magnoliid *Persea americana* (avocado) and *Amborella trichopoda*, which is sister to the rest of the angiosperms. The genomes were firstly curated to filter out truncated reading frames and sequences showing similarity to transposable elements, resulting in genomes ranging from 18,254 genes in Moringa to 37,385 genes in maize (Table 1). To obtain the orthogroup classification, we compared the 295,716 total final sequences to each other. On the basis of this comparison, 235,368 out of those sequences (i.e., 79.6%) could be classified into a total of 17,998 orthogroups containing at least two genes (Table 1). The rest, 60,347, including 1307 exclusive to Moringa, were classified as unassigned to any orthogroup, corresponding to singleton orphan sequences (Table 1). The remaining 16,947 genes in the Moringa genome, i.e., nearly 93% of the total, were assigned into 11,372 orthogroups (Table 1). In turn, only four orthogroups, grouping together 37 genes, are exclusive to Moringa, which represents the second species, only after *L. purpureus*, with the smallest percentage of genes found in species-specific orthogroups.

**Table 1:** Summary of results from Orthofinder classification in orthogroups in the genome of Moringa and 10 other flowering plant species.

	*Arabidopsis thaliana (Ath)*	*Amborella trichopoda (Atr)*	*Faidherbia albida (Fal)*	*Lablab purpureus (Lpu)*	*Moringa oleifera (Mob)*	*Oryza sativa ssp. Japonica (Osj)*	*Persea americana cv. Hass (Pah)*	*Sclerocarya birrea (Sbi)*	*Vigna subterránea (Vsu)*	*Vitis vinifera (Vvi)*	*Zea mays (Zma)*
Number of genes	27,371	26,120	28,924	20,893	18,254	35,687	24,596	18,911	31,577	25,998	37,385
Number of genes in orthogroups	22,317	17,206	23,668	19,466	16,947	23,951	20,236	16,916	25,878	20,100	28,683
Number of unassigned genes	5,054	8,914	5,256	1,427	1,307	11,736	4,360	1,995	5,699	5,898	8,702
Percentage of genes in orthogroups	81.5	65.9	81.8	93.2	92.8	67.1	82.3	89.5	82	77.3	76.7
Percentage of unassigned genes	18.5	34.1	18.2	6.8	7.2	32.9	17.7	10.5	18	22.7	23.3
Number of orthogroups containing species	11,649	11,637	12,492	11,647	11,372	13,211	11,451	10,764	13,242	11,845	13,512
Percentage of orthogroups containing species	64.7	64.7	69.4	64.7	63.2	73.4	63.6	59.8	73.6	65.8	75.1
Number of species-specific orthogroups	50	45	44	7	4	41	29	17	40	21	72
Number of genes in species-specific orthogroups	428	289	202	26	37	189	135	84	198	88	275
Percentage of genes in species-specific orthogroups	1.6	1.1	0.7	0.1	0.2	0.5	0.5	0.4	0.6	0.3	0.7

**Figure 1:**
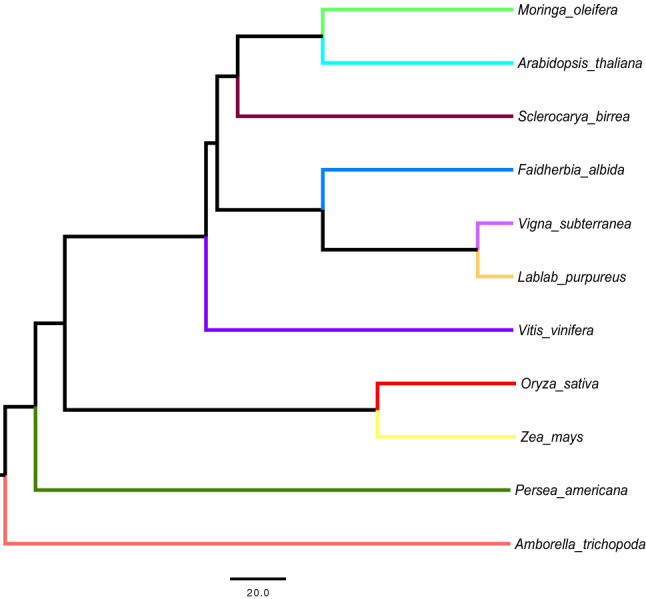
Evolutionary tree of *Moringa* and 10 angiosperm species examined in this study. Branch topologies and divergence times in the tree are based on estimates from TimeTree^27^. Branch lengths reflect evolutionary time (in millions of years). Previous works resulted in conflicting evolutionary hypotheses placing magnoliids as forming a sister clade either to eudicots or to monocots, or as a sister clade with respect to the supergroup formed by all eudicot and monocot plants combined^28–30^. In this tree we used the latter hypothesis to branch the Magnoliid representative avocado (*Persea Americana*).

In addition, to test evolutionary hypothesis of significant gene family expansion and contraction in the Moringa lineage, we constructed an ultrametric phylogenetic tree depicting the evolutionary relationships among Moringa and the remaining 10 species (Fig. 1). The resulting 11-species tree and gene family classification in 17,998 orthogroup were then used to evaluate the fit of different ML models of gene family evolution implemented in BadiRate^26^. For a total of 419 gene families, the model estimated significantly different gene gain and death rates in the Moringa branch compared to the rest of branches in the tree provided the best fit to the data according to the AIC test (Supplementary Table S2). These included 318 identified as significantly contracted, of which 296 would have lost all genes while the remaining 22 group a total of 47 genes, and 101 families, grouping a total of 957 genes, found as significantly expanded.

### Functional categorization of contracted gene families

To get a first insight of gene and gene functions that may have been preferentially lost in the compact Moringa genome, we used our functional annotation of the genome with GO terms. Of the 318 families identified as significantly contracted in Moringa, only 22 have retained any genes, grouping a total of 47 (Supplementary Table S2). 45 out of these 47 genes were annotated with at least one GO term, up to a total of 235 (Supplementary Table S3). Of the eight different GO terms significantly overrepresented among contracted families, five were found annotating orthogroup OG0000010 (Supplementary Table S3), which contains eight transcription factors in Moringa and 13 to 23 in the rest of species belonging to the type II MIKC family of MADS-box transcription factors, known to control key processes of plant development including vegetative growth, regulation of flowering time and floral/fruit patterning^31^.

Other interesting instances of Moringa-specific contractions occurred in orthogroups OG0000594 and OG0000019 (Supplementary Table S3). The former corresponds to the Phenylalanine N-monooxygenase oxidoreductase gene family, a cytochrome P450 enzyme which converts L-phenylalanine and Tryptophan into an oxime in the first step of indole glucosinolate biosynthesis, which has lost all members in Moringa^32,33^. The latter encodes for ST5A/SOT16, an aromatic desulfoglucosinolate sulfotransferase involved in the last biosynthetic step of active benzyl glucosinolates from their desulphoglucosinolate relatives^34^, which has lost all its members in Moringa but one, while conserving from six to 33 genes in the rest of species.

### Functional categorization of expanded gene families

Similarly, to describe the functional gene space gained by the Moringa genome, we functionally categorize the set of genes belonging to expanded families by means of GO terms. Of the 957 genes belonging to expanded gene families, 848 could be annotated with at least one GO term. The 848 annotated genes summed up a total of 4,057 GO terms, which represents an average of 4.78 GO terms per annotated gene.

Of the 5436 different GO terms found in the Moringa genome, 81 were found as differentially represented among expanded gene families, including 68 overrepresented (Supplementary Table S4). Most overrepresented GO terms corresponded to plastid and, especially, chloroplast associated functions. For example, all 15 GO terms in the cellular component class are related to plastids, including “chloroplast” and “chloroplast thylakoid membrane”, the two most strongly overrepresented, “plastid” or “photosystem” (Supplementary Table S4). Similarly, 23 of the 25 GO terms in the biological process class are occurring in chloroplasts, including “ATP synthesis coupled proton transport”, “photosynthesis”, “reductive pentose-phosphate cycle” or “photorespiration” (Supplementary Table S4). Finally, 22 of the 28 GO terms of the molecular function class were related to enzymatic activities or biochemical processes associated with chloroplasts, among which “proton-transporting ATP synthase activity, rotational mechanism”, “electron transporter, transferring electrons within the cyclic electron transport pathway of photosynthesis activity” or “ribulose-bisphosphate carboxylase activity” (Supplementary Table S4).

We further examine the most significantly expanded orthogroups in Moringa. Among them, we found many orthogroups clustering with Arabidopsis plastid encoded genes, including, OG0000125, annotated as ribulose-1,5-bisphosphate carboxylase/oxygenase (RuBisCO) large subunit (RBCL), OG0000374, encoding for the CP47 subunit of the photosystem II reaction center, OG0003080, corresponding to the plastidic NADH dehydrogenase enzymatic activity, OG0000842, annotated as encoding for the beta subunit of the plastidic acetyl-CoA carboxylase carboxyltransferase, OG0000207, conforming the RNA polymerase beta' subunit-2 involved in plastid transcription machinery, or OG0003554, annotated as RF1/YCF1, a group of proteins highly variable both in length and number of transmembrane regions which play a key role in the plastid protein import machinery^35^ and has 11 members in Moringa, zero in most species and five in Arabidopsis (Supplementary Table S4).

Indeed, up to 27 out of the 101 families identified as expanded corresponded to orthogroups that included at least one Arabidopsis orthologous gene encoded by the plastid genome. These 27 families clustered a total of 457 Moringa genes, i.e., about 48% of the total 957 genes belonging to expanded families. Other expanded families were also of probable plastid origin but were not grouped in orthogroups together with their Arabidopsis plastid orthologues. For example, we found up to five gene families annotated as chloroplast RF21/YCF2 proteins, including OG0000242, OG0001443, OG0010929, OG0013688 and OG0012456, highly expanded in Moringa with a total of 69 genes for zero to 19 in the rest of species (Supplementary Table S4). *YCF2* are among the largest genes found in the Arabidopsis plastid genome and encode for proteins with ATPase activity, which participates in the formation of a 2-MD heteromeric AAA-ATPase complex that associates with the plastid protein translocon complex formed by YCF1 and functions as the import motor^36^.

Although most of the enrichment in chloroplast functions resulted from the specific expansion of genes originally encoded by the plastid genome and relocated to the nuclear genome, a few nuclear gene families involved in plastid functions could also be found as expanded. These included OG0001392, encoding for different protein components of the small subunits of the plastid ribosome, OG0003289, grouping four genes in Moringa and 1–2 in the remaining species, including the Arabidopsis enzyme Lycopene β-cyclase, involved in cyclic carotenoid and xanthophyill biosynthesis^37^, OG0004457, clustering three Moringa genes and one-two in the rest of species, annotated as FLUORESCENT IN BLUE LIGHT (FLU), a protein involved in chlorophyll biosynthesis or OG0004658, encoding for the B subunit of the plastidial ATP synthase (Supplementary Table S4).

Among the expanded families formed by genes non directly related to plastid or chloroplast functions, we also found several enrichments in specific enzymatic activities of the secondary metabolism. Among these, the most significantly enriched GO term was “S-adenosylmethionine-dependent methyltransferase activity”, with 16 genes in Moringa distributed in two expanded families (OG0000577 and OG0006004) (Supplementary Table S4). This enzymatic activity transfers a methyl group to a wide range of substrates, including several low molecular weight metabolites acting as plant hormones, and is key in phenylpropanoid, flavonoid and glucosinolate plant metabolic pathways^38,39^. Another notable expansion was found in orthogroup OG0007231, which contains seven genes in Moringa, five in Arabidopsis and none in the rest of species, encoding for members of the large family of GDSL-type esterases/lipases, formed by hydrolytic enzymes with multifunctional properties such as broad substrate specificity^40^, including Arabidopsis epithiospecifier modifier 1 (ESM1), which represses nitrile formation and favors isothiocyanate production during glucosinolate hydrolysis^41,42^.

### Sequence and phylogenetic analysis of the OG0000125 orthogroup, encoding for the *RBCL* gene family

**Figure 2:**
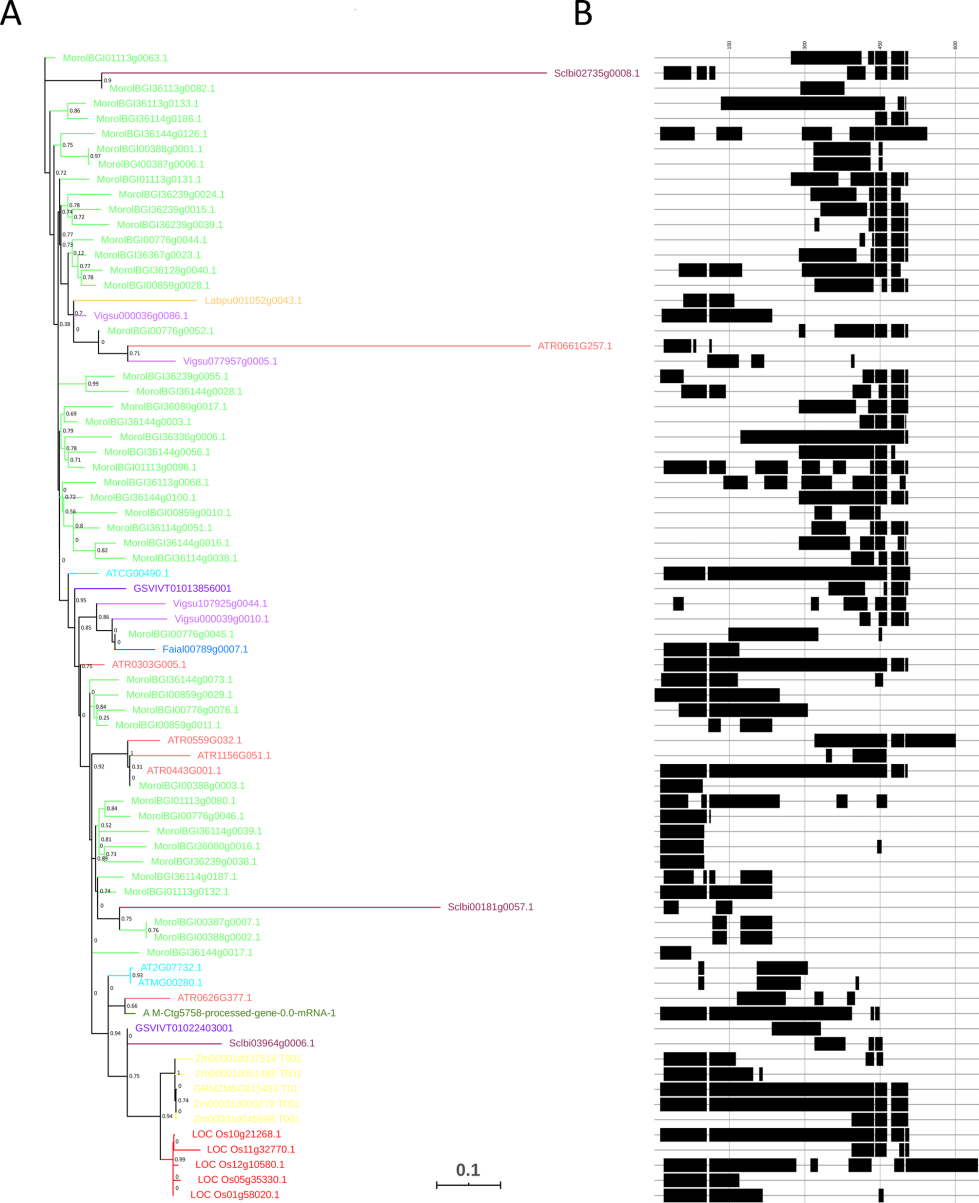
Unrooted ML phylogenetic tree and multiple protein sequence alignment of 76 *RBCL* genes from Moringa and 10 angiosperm species. (**A**) The ML tree is based on *RBCL* nucleotide sequences and is drawn to scale, with branch lengths proportional to evolutionary distances between nodes. The scale bar indicates the estimated number of nucleotide substitutions per site. Branches in the tree are coloured according to plant species in Fig. 1. Statistical support values (posterior probabilities from aLRT tests) for clades are shown next to the corresponding nodes. (**B**) Schematic representation of the multiple alignment of 76 RBCL protein sequences. Regions of the alignment are indicated by black boxes, and gaps by black lines. Scale is represented by vertical black lines located 150 positions apart in the alignment.

Next, we explored the evolutionary origin and diversification of multiple copies of plastid duplicated genes belonging to expanded gene families. For this purpose, we selected the OG0000125 orthogroup, corresponding to the *RBCL* gene family, highly expanded in Moringa with 45 genes, and present in the rest of species with one to six members (Supplementary Table S2). Of the three Arabidopsis *RBCL* genes: only one ATCG00490.1 encoded for the functional, full length (479 amino acids) plastid-encoded large subunit of ribulose-1,5-bisphosphate carboxylase/oxygenase (RuBisCo). The two additional copies, ATMG00280.1 and AT2G07732.1, are encoded by the mitochondrial and nuclear genomes and correspond to shorter proteins of 110 and 116 amino acids long, respectively. The multiple alignment of the 76 total protein sequences conforming orthogroup OG0000125, varying in length from 63 to 491 amino acids, revealed a similar pattern (Fig. 2B). Although in several species full-length RBCL proteins could be detected, most members of the family corresponded to shorter protein sequences. This was the case of Moringa, whose 45 genes encoded for protein sequences ranging from 63 to 344 amino acids.

In an attempt to describe the evolutionary history of the 76 sequences conforming orthogroup OG0000125, we performed a ML phylogenetic analysis based on the codon alignment. The resulting tree, represented in Fig. 2A, shows extremely short branches and low statistical support, particularly at internal nodes, likely because of the extremely short, or even absent, aligned regions in some pairwise sequence comparisons, together with the high level of sequence conservation. Most Moringa *RBCL* sequences appeared clustering together in the tree, forming clades of 3 to 6 genes (Fig. 2A). Additional clustering might be eventually obscured by highly diverged sequences causing long branch artifacts in the tree. Despite the low phylogenetic signal of the alignment to solve deep evolutionary relationships, the general picture emerging from the tree reflects recent events of plastid gene duplication at the origin of the observed Moringa-specific expansion.

### Synteny analysis of the Moringa plastid and nuclear genomic regions containing the expanded *RBCL* gene family

**Figure 3:**
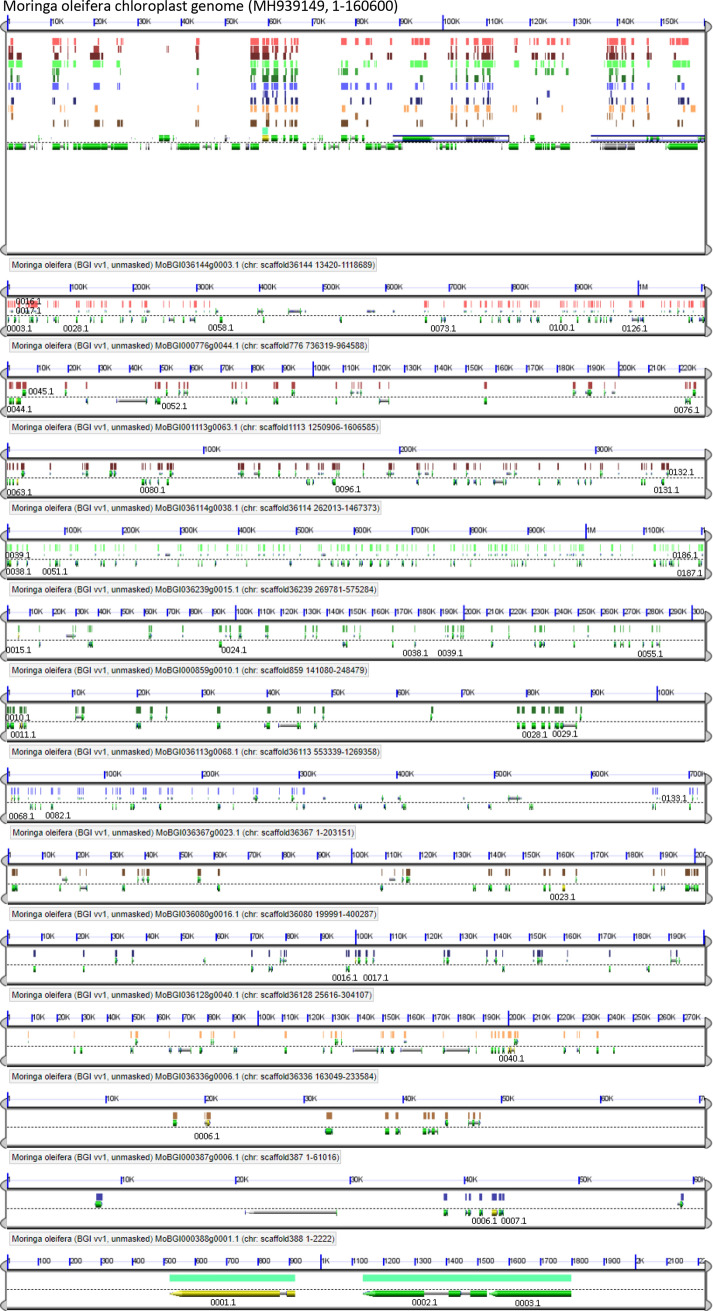
Microynteny analysis of the Moringa chloroplast genome and 13 genomic regions containing *RBCL* genes. Each subpanel represents a genomic region, with gene models on both strands shown above and below the dashed line, respectively. Ids of Moringa *RBCL* gene models are indicated at their corresponding genomic locations. Pairwise genomic comparisons were performed using the Moringa chloroplast genome, located on top, as reference. High-scoring sequence pairs (HSPs) between protein-coding sequences are marked by short coloured vertical bars on top of the corresponding gene models in the Moringa chloroplast genome. Collinear series of HSPs across genomic regions indicates a syntenic relationship between the regions concerned.

To gain insights into the mechanisms at the origin of the recurrent relocation of plastid genes in the nuclear genome of Moringa, we examined the genomic regions containing *RBCL* duplicated genes. The 45 *RBCL* genes in Moringa form clusters of one up to eight genes along 13 non-contig regions or scaffolds of the genome, ranging in size from to 2222 to 3,436,153 base pairs (bp). One way to determine whether the plastid genes were acquired through the duplication of short regions of the plastid genome containing single genes or gene fragments or larger regions containing sets of genes, is to examine for the occurrence of synteny. Synteny between genomic regions, i.e., the colinear arrangement of putative homoeologous genes, indicates that they have evolved from a common ancestral genomic region. We subjected the 13 *RBCL* genomic regions and the plastid genome of Moringa to microsynteny analysis using the GeVo tool from the CoGe platform. Results, represented in Fig. 3, showed at least 11 out of the 13 *RBCL* genomic regions, displaying strong syntenic relationships with the plastid genome, with the number of collinear genes highly similar in most comparisons. Some duplicated *RBCL* genes were located in consecutive positions of the genome, suggesting either multiple insertions of the original plastid *RBCL* gene, or eventually tandem duplication following insertion. In the large scaffold 36,144, synteny could be observed with different regions, suggesting multiple insertion sites of large fragments of the plastid genome. For the two shorter scaffolds, containing only a few genes, synteny could not be ascertained. In conclusion, the strong syntenic signal shown by most *RBCL* genomic regions reveals that plastid gene duplicates originated through insertions of large regions of the plastid genome.

### Analysis of plastid DNA insertions in the Moringa nuclear genome

To characterize the number and size distribution of plastid DNA insertions in the nuclear genome, we used BLASTN to scan the entire Moringa genome using the sequence of the Moringa plastid genome as a query. A total of 10,919 local alignments were formed with at least 896 scaffolds; of these, only 115 showed more than 2000 total aligned bp. The total aligned region between the plastid genome and the nuclear genome summed up a total of 10,215,907 bp, which represents a 4.71% of the 216,759,177 bp of the assembled genome. The size distribution of plastid DNA insertions in the nuclear genome shows a non-normal right-skewed unimodal distribution ranging from 34 to 24,143 bp, with a mean and a median of 936 and 195, respectively (Supplementary Fig. S1). In order to validate these results and discard any assembly bias that could be at the origin of such high fraction of plastid DNA found in the Moringa nuclear genome, we repeated this analysis using a different version of the Moringa genome^43^. 12,807,394 bp out of 289,241,074 total bp in the assembled nuclear genome, i.e., a 4.43%, was conformed of plastid DNA, pretty close to the 4.71% found in the version of the genome used in our analysis.

**Figure 4:**
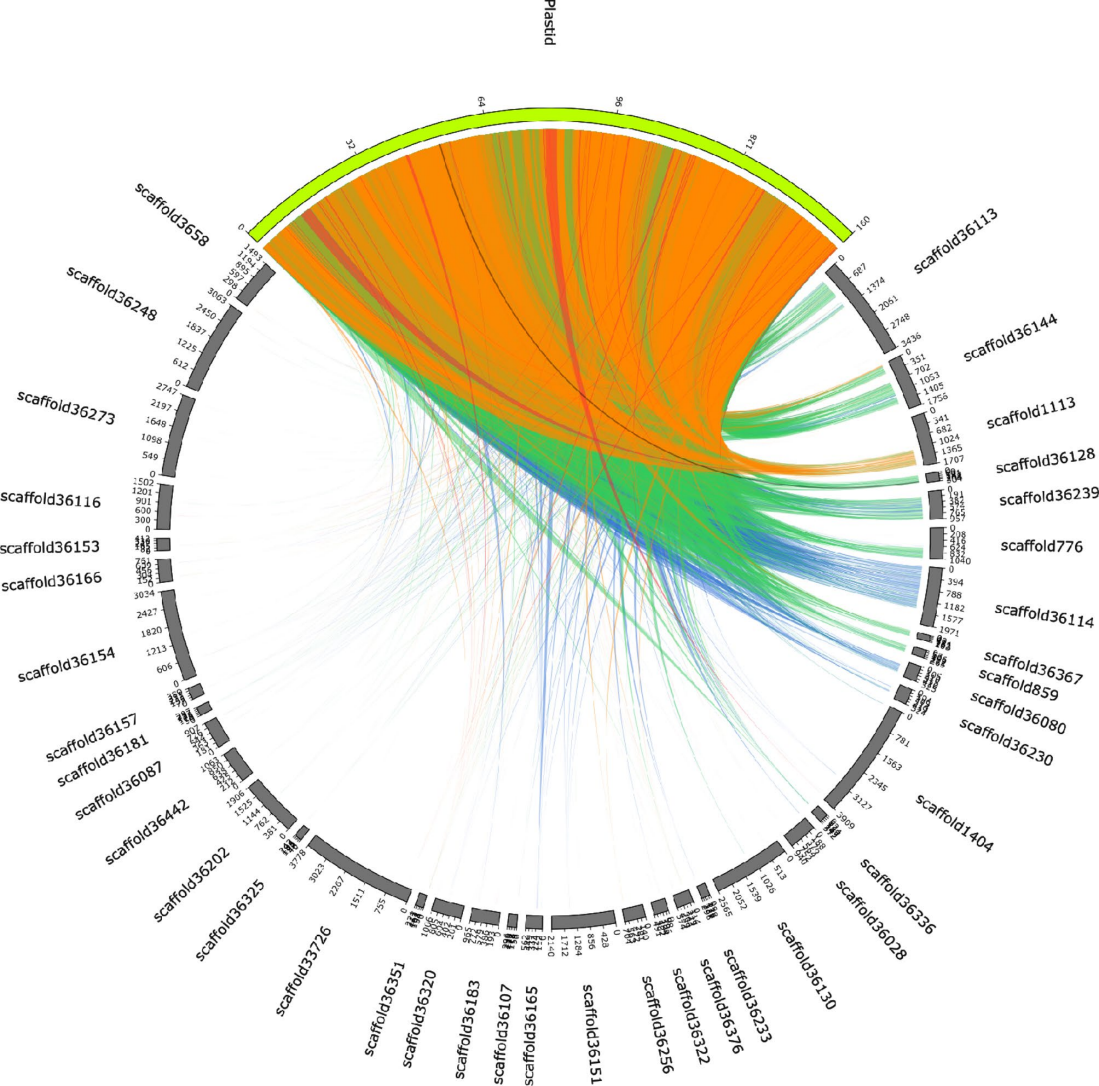
Circos plot representation of plastid DNA insertions in the Moringa nuclear genome. Nuclear genome scaffolds and the plastid genome are represented as dark grey and green filled blocks, respectively, forming a circle. Only the 39 nuclear genome scaffolds with a length equal or higher than that of the plastid genome (160,600 base pairs) and returning BLASTN alignments between them with at least 90% of sequence identity over a region of minimum 2000 bp are shown. The block corresponding to the plastid genome is located at 12 o'clock, and the 39 nuclear genome scaffolds are arranged clockwise according to the total sequence length involved in BLASTN alignments with plastid DNA. Nuclear genome scaffolds are drawn to scale, with lengths proportional to size, while the plastid genome has been upscaled to occupy a quarter of the image circumference; in each case, the scale represents 1,000 base pairs. Local BLASTN alignments are represented as ribbons. Ribbons are colored according to the percentage of sequence identity of the local alignment using the (score − min)/(max − min) ratio colouring with blue ≤ 0.25, green ≤ 0.50, orange ≤ 0.75, red > 0.75.

Results for the 39 scaffolds showing the best BLASTN alignments with the plastid genome (i.e., with a length equal or higher than that of the plastid genome—160,600 base pairs—and returning BLAST alignments with at least 90% of sequence identity over a region of minimum 2000 bp) were graphically represented as a circular Circos plot diagram. Scaffolds are arranged in the plot clockwise according to the total length of the aligned region, while the plastid genome is located centered at the upper part of the plot (Fig. 4). Some scaffolds with the largest aligned regions spanned several times the size of the plastid genome, reflecting multiple insertions of large regions. Among these scaffolds we found the ones containing the *RBCL* genes (Fig. 3). Insertion sites are found contiguously along the nuclear genome, but can also be found at distant locations, e.g., scaffold 36,144 (Fig. 4). The remaining scaffolds mostly showed insertions of short regions of plastid DNA (Fig. 4).

### Modeling genome duplications in Moringa and Arabidopsis

**Figure 5:**
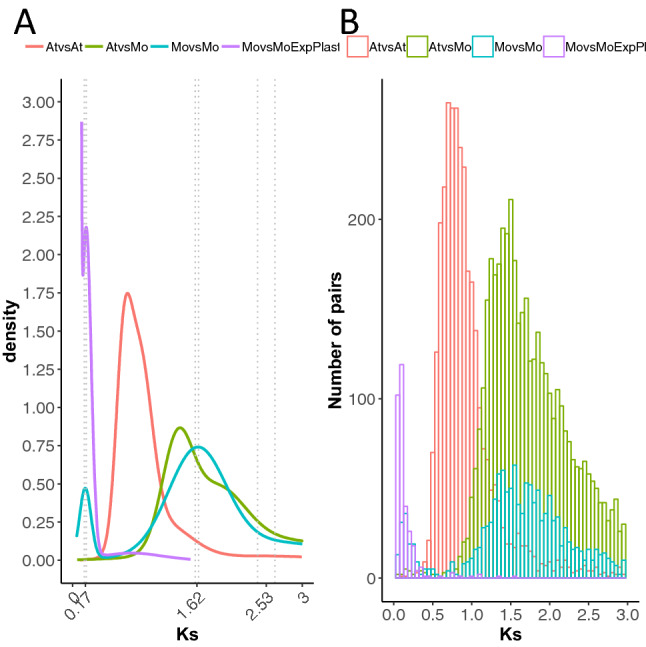
Modeling genome duplications in Moringa and Arabidopsis. Density plots from fitting Gaussian mixture models to *K*s distributions estimated from pairs of syntenic paralogues within the Moringa and Arabidopsis genomes, of syntenic orthologues between both genomes as well as the duplicated genes belonging to the 27 plastid gene families found as expanded by the BaidRate analysis. Vertical dashed bars indicate 95% confidence intervals around the means of the peaks detected by mclust.

The transfer of plastid DNA and subsequent integration at different locations of the nuclear genome might have occurred simultaneously at a single time point, or continuously throughout evolutionary time. Although both scenarios are not mutually incompatible, the former scenario of episodic recurrent plastid genome duplication should leave a single peak in the distribution of synonymous substitutions (Ks) between duplicated gene pairs, independent from other peaks corresponding to putative nuclear WGD. We modeled the distribution of Ks between syntenic paralogues detected in the Moringa and Arabidopsis genomes, as well as of syntenic orthologues between both genomes (Fig. 5B). In the Moringa genome, we detected 242 syntenic blocks comprising 2612 paralog pairs. In the Arabidopsis genome, 283 syntenic blocks comprising 6489 paralog pairs were identified. Between Moringa and Arabidopsis, 5221 pairs of syntenic orthologues clustering into 356 syntenic genomic blocks were found. In order to identify peaks in the Ks distributions that could be indicative of episodic genome duplication, we fitted Gaussian mixture models by using the R library mclust. For syntenic Moringa paralogs three peaks were detected (Fig. 5A). The main one is centered at ~ 1.62 and matches a secondary peak also detected among Arabidopsis paralogs. According to the relative location of this peak with respect to the main peak formed by syntenic orthologues between Moringa and Arabidopsis, which marks the divergence time between the two species, it might correspond to the Gamma WGD event shared by all eudicot plants^20^. To the right of this peak there is an older, secondary peak centered at 2.53, which might in turn correspond to the Angiosperm-specific Epsilon WGD event^19^ (Fig. 5A). To the left of the major peak there is a much younger, secondary peak centered at 0.17, specific to Moringa and also located to the left of the major peak detected for Arabidopsis syntenic paralogues, which likely corresponds to the alpha Brassicaceae-specific WGD event^44^ (Fig. 5A). This younger peak contained 129 pairs of duplicates, of which 126 were included among the 457 genes contained in the Moringa expanded plastid families. When we used the alternative R library mixtools to fit Gaussian mixture models to Moringa paralogs, we also obtained three peaks with similar means of 0.164, 1.623 and 2.496, within the 95% confidence interval of mclust estimates (Fig. 5A).

We similarly modeled the distribution of Ks for all pairs of Moringa gene duplicates present in the 27 plastid gene families specifically expanded in Moringa applying a method to correct for redundancy in duplication nodes and saturation of Ks estimates^45^ (Fig. 5B). Using mclust, a peak of 0.177, within the 95% confidence interval of the younger secondary peak detected for Moringa syntenic paralogs, could be detected over the L-shaped curve likely formed by Ks estimates of recent tandem duplicates and/or plastid DNA insertions (Fig. 5A). A single peak was also detected by mixtools centered at 0.174. We concluded that a significant part of the expansion in plastid genes occurred recently through simultaneous episodes of duplication of large regions of the plastid genome and subsequent relocation to the nuclear genome.

## Discussion

Evolutionary realization of endosymbiosis requires that most of the genes for plastid functions originally encoded in the pro-plastid genome are transferred to the nucleus and their products retargeted to their ancestral compartment; as a result, pro-plastid genomes have lost most of their original genes ^46^. Although in most eukaryotes transfer of functional genes is now rare or has ceased altogether, plant organelle DNAs are still being ubiquitously and continuously transferred to the nucleus and inserted in nuclear genomes^25^ much more frequently than generally believed^47^. Most recent events of plastid DNA transfer involve sequences less than 1 kb in length. Only a few ones involving larger regions of the plastid genome have been documented. For example, a transfer event of plastid DNA to the nucleus was estimated to occur recently in japonica rice, after divergence with the indica lineage and the closely related species *O. rufipogon*, which resulted in two sequences 99.77% identical along 97% of the plastid genome^48^. In most species, plastid DNA represents only a small fraction of less than 1% of the nuclear genome. Very few species have more than 1%, including *Ziziphus jujuba,* whose genome is composed of up to 1.49% of repeated insertions of small fragments of plastid DNA^49^.

Our comparative genome wide evolutionary analysis of gene family expansions and contractions in the genome of Moringa and 10 other flowering plant species reveal the strong expansion of plastid and chloroplast related genes and gene functions. Rather than resulting from duplications of nuclear genes, the expansion observed seemed to result from the repeated transfer of plastid DNA to the nucleus and subsequent integration at several locations of the nuclear genome; in other words, the duplication of plastid genes into the nuclear genome. Most plastid duplicated genes would thus have originated through a recent burst of duplications involving large regions of the plastid genome, as revealed (i) the microsynteny analysis of the Moringa genomic regions containing expanded *RBCL* genes and its plastid genome, and (ii) the peak in the distribution of Ks values shared by syntenic paralogues and duplicates belonging to Moringa-expanded plastid gene families. Although the simultaneous duplication of large regions of the plastid genome at specific locations of the nuclear genome was responsible of most plastid DNA detected, abundant insertions of very diverse sizes were also found in many different locations of the nuclear genome. Altogether, the fraction of plastid DNA found in the Moringa nuclear genome represented 4.71%, the largest so far reported for a plant genome^49^.

Although the origins and the evolutionary paths of insertions of organelle DNA into the nuclear genome are probably diverse, they involve double-stranded breaks and DNA damage and thus are potentially mutagenic^25,50^. Furthermore, the uncontrolled proliferative insertion of organelle DNA might lead to the unnecessary obesity of the nuclear genome^51^. Therefore, plastid DNA insertions are expected to be neutral or eventually deleterious and selected against^25,50^. One question that immediately arises is about the selective forces that may have operated on the Moringa lineage promoting the repeated transfer of massive amounts of plastid DNA to the nucleus. DNA migration from chloroplasts to the nucleus has been shown to be markedly increased by different forms of biotic and abiotic stress, notably mild heat stress^50,52^. Furthermore, disruption of organelle membranes which can occur during cell stress and gametogenesis also facilitates DNA to escape from organelles and makes it more accessible for illegitimate uptake via the nuclear import machinery^25,53^. It cannot be discarded that the Moringa lineage had been subjected to specific stressful conditions recently during its evolutionary story promoting the observed burst of plastid DNA insertions. Indeed, domestication from the low regions of the Himalayas in northwest India, where the plant is believed to originate and mean annual precipitations exceed 1100 mm, to adapt to tropical and sub-tropical areas around the world where its culture has expanded, is expected to have occurred through the selection of varieties better adapted to drier and hotter environments^5,54^. Moringa has been reported to successfully cope with multiple stresses, particularly water deficit and UVB radiation, which result in the impairment of the photosynthetic apparatus, increase in reactive oxygen species and reduced plant productivity, by adjusting carbon metabolism and antioxidant battery^3^ and producing specific secondary metabolites, especially isoprene and flavonoids^54^.

Plastid genes are usually inactive upon arrival into the nuclear genome because they lack the regulatory motifs required for proper gene expression. Most of them are thus expected to evolve as pseudogenes or non-coding sequences^25,49^. Studies performed on Arabidopsis and rice reveal organellar DNA insertions decay over evolutionary time into smaller fragments with more divergent sequences^53^. This decay is expected to occur quickly; in rice it is estimated that 80% of plastid DNA insertions are eliminated from the nuclear genome within a million years as a result of rapid fragmentation and vigorous shuffling^55^. This fragmented pattern reported for plastid genes after relocation to the nucleus is also observed here for the highly expanded *RBCL* gene family in Moringa. Although the molecular mechanisms promoting the erosion and fragmentation of recently inserted plastid DNA are not fully elucidated, they probably involve the insertion of transposable elements and other DNA sequences unrelated to organelle DNA^56^. It must be noted, however, that all *RBCL* genes in Moringa corresponded to open reading frames with very conserved sequences, suggesting they might be actually expressed into proteins. Their corresponding non-plastid orthologues in Arabidopsis*, ATMG00280.1 and AT2G07732.1*, are actually expressed and can be detected across different expression experiments (https://www.arabidopsis.org/ and https://bar.utoronto.ca/eplant/). The expression of multiple copies of fragmented *RBCL* genes, if properly retargeted to the plastid, might impact the structure and function of the RuBisCo multiprotein complex^57^, encoded by both plastid and nuclear genes. Moreover, in certain occasions newly arrived organellar genes have been reported to gain expression capabilities in the nucleus or to reshape nuclear genes by adding extra coding sequences, both contributing to enhance genetic diversity and opportunity for the origin of new gene functions ^49,58^. The repeated transfer of large chunks of plastid DNA to the nuclear genome may have thus provided the plant with a formidable source of genetic material to modify pre-existing gene functions and/or acquire novel ones.

We also identified several Moringa-specific expansions and contractions in gene families involved in secondary metabolism, which might be at the origin of the notable production of species-specific bioactive natural compounds by the plant^4,7^, especially glucosinolates^9^. Moringa produces two major glucosinolates, glucomoringin and glucosoonjnain, of which the latter is apparently responsible of most of the bitter harsh taste of leaves. Interestingly, a recent work reported that domestic accessions of Moringa showed higher levels of the former and lower levels of the latter, while the opposite was true for wild type accessions, suggesting that domestication of Moringa may have selected against glucosoonjnain and better taste variants^8^. A wide range of medicinal properties have been attributed to glucosinolate products, and in special to their cognate isothiocyanates resulting from hydrolysis through the action of myrosinase enzymes^9^. Although myrosinase activity was not found to be significantly higher in domesticated plants regarding wild type populations^8^, it is interesting to note that orthogroup OG0010827, corresponding to the myrosinase family, was found expanded in Moringa, although not significantly, with 18 genes for 15 in Arabidopsis and four to 16 in the remaining species (Supplementary Table S2). The classification and functional annotation of gene families involved in different aspects of glucosinolate and other secondary metabolic biosynthetic pathways in Moringa presented here, provides a solid framework for further genetic and biochemical studies of bioactive compound production.

In summary, our exploration of the adaptive landscape of the Moringa genome reveals interesting lineage-specific expansions and contractions in specific gene families and functions and highlights the importance of performing thorough genome wide evolutionary analysis of neglected understudied crops to provide with candidate genes and molecular markers potentially at the origin of interesting biological, agronomical or pharmaceutical properties. Furthermore, Moringa emerges as a model plant organism to study the molecular evolutionary mechanisms underlying plastid gene duplication and its potential impact on the evolution of nuclear genome structure and function.

## Experimental procedures

### Orthogroup/gene family classification

The sequences from the complete proteomes of Moringa and 10 other plant species (Supplementary Table S1), were firstly compared all-against-all using Diamond^59^ and then classified into orthogroups using the clustering algorithm implemented in OrthoFinder v2.3.3^60^ under the default settings. Prior to the analysis, the proteomes were scanned and truncated proteins and sequences showing significant similarity to transposable elements, as resulting from BLASTX searches against the RepBase v23.08 database (E-value < 10E-5, bit score ≥ 45)^61^, were filtered out.

### Modeling gene family evolutionary dynamics

To model the evolution of gene families resulting from a comparison of the Moringa genome and 10 other plant species, we applied the ML gain and death (GD) stochastic models implemented in the BadiRate program^26^. BadiRate allows testing biologically relevant evolutionary hypothesis on the data using a species phylogenetic tree. A phylogenetic tree depicting the evolutionary relationships among the 11 species was manually reconstructed using the topology and the median divergence times reported in TimeTree^27^.

Three competing evolutionary models can be tested in BadiRate. First, the Global Ratio model, which estimates the same gain and death rates over all branches in the tree and corresponds to gene families that have remained stable over evolution in terms of number of genes. Second, under the Free Ratio model, gain and death rates are estimated independently for every branch in the tree, fitting well with families that have evolved stochastically. Finally, the Branch Ratio models estimates distinct gain and death rates for the branch corresponding to our species of interest (i.e., Moringa) and for the rest of branches in the tree, and may be indicative of lineage-specific gene family expansion or contraction.

For each orthogroup in our 11 species genome wide classification, we ran five replicates of each independent evolutionary model, and the resulting log likelihoods were compared by means of AIC tests^62^. When the result of the AIC test for the best model was 2.7 times higher than that of the second-best model, the former was selected as providing a significant best fit.

### Functional annotation of the Moringa genome

We performed the functional annotation with Gene Ontology (GO) terms of the 18,254 proteins encoded by the Moringa genome (after removing truncated or repetitive sequences) using the BLAST2GO program^63^, based on homology inferences with proteins from other species obtained through BLAST, the appearance of INTERPRO protein functional domains, and the EC enzyme codes represented in the KEGG biochemical pathways. The following parameters were used: fast-BLASTP searches were performed against the nr database using an HSP cutoff length of 33, report 20 hits, maximum E-value 1 E−10, followed by mapping and annotation of Moringa proteins with the GO terms identified in BLAST hits using the default settings. GO terms for each protein were further confirmed or, where appropriate, expanded with those associated with the INTERPRO domains detected by INTERPROSCAN. As a result, a total of 64,408 GO terms were assigned to 15,611 of the 18,254 genes present in the Moringa genome, i.e., a substantial fraction of the Moringa genes, 85.5%, had at least one GO term.

Analysis of statistical significance of differential distributions of GO terms between subsets of genes and all genes present in the genome was performed by means of Fisher's exact tests^64^. To control for multiple hypotheses testing, the resulting P values were corrected according to the Bonferroni test^65^, and those < 0.05 were considered significant.

### Identification of syntenic homologues and microsynteny analysis

Sets of syntenic paralogues in the genomes of Moringa and Arabidopsis and of orthologues between both genomes (defined by series of collinearly arranged putative homologous genes) were extracted using the SynMap tool from the CoGe platform^66^, with default parameters and the Quota Align algorithm to merge syntenic blocks^67^. We used the default settings to define the minimum number of collinear genes for two regions to be called syntenic.

Microsynteny analyses were performed using the GEvo tool from CoGe. Non-coding regions were masked to include only protein-coding sequences.

### Detection of plastid DNA insertions in the Moringa nuclear genome

Insertions of plastid DNA in the Moringa nuclear genome were detected using the BLASTN local alignment tool from the BLAST+ program package (version 2.10.0+)^68^. The Moringa chloroplast genome sequence was used as query (NCBI GenBank Accession Number MH939149)^69^ and the nuclear genome sequence used in this work^2^, together with a second one published elsewhere^43^, used as databases in separate scans. The parameters were as follows: -max_target_seqs 1000, -dust no, e-value threshold of 1e−5, mismatch penalty of – 2, and word size of 9. Results in terms of sequence similarity were represented as circular plots, constructed using Circos version 0.69−8^70^ and the Circoletto tool with the settings --score2colour id to color ribbons using the percentage of sequence identity as score and --scoreratio2colour minmax to color ribbons according to the formula (score − min)/(max − min), which should give more colour range especially for percent identity^71^.

### Gaussian mixture model analysis of *K*s distributions

Codon sequences were aligned with PRANK v.140603 using the settings -codon, to align coding DNA based on the empirical codon model^72,73^, and -F, i.e., always skipping insertions^72^. Estimates of Ks were obtained using the CODEML program^74^ from the PAML package (v4.8)^75^ on the basis of codon sequence alignments. We used the GY model with stationary codon frequencies empirically estimated by the F3 × 4 model. To avoid suboptimal estimates because of ML entrapment in local maxima, five replicates were run for each pairwise sequence Ks estimation and the best one was selected.

Estimates of Ks were computed for two different sets of duplicate gene pairs. First, for syntenic orthologs and paralogs in Arabidopsis and Moringa identified through SynMap. Second, for all pairwise alignments among genes belonging to a subset of gene families from our 11-species classification identified in this study as expanded in Moringa. In order to correct for redundancy of Ks values in the latter (a gene family of n members produces n(n-1)/2 pairwise Ks estimates for n-1 retained duplication events), we followed an approach based on Vanneste et al., 2013^45^. Briefly, for each gene family, a phylogenetic tree was constructed using PhyML v3.1^76^ under default settings, based on the multiple alignment of protein sequences obtained using MUSCLE v3.8.31^77^, further edited using trimAl v1.2rev59 with the option -automated1^78^. Phylogenetic trees were subsequently midpoint rooted using the biotree -m tool from the Bio-BPWrapper-1.13 PERL library. Starting on the root, every tree is split recursively into the resulting child clades until all Ks pairwise estimates within members of each subclade do not exceed 5. For each duplication node in the resulting subclade, all m (where m is the number of duplication events) Ks estimates between the two child clades are added to the Ks distribution with a weight of 1/m, so that the weights of all Ks estimates for a single duplication event summed to one.

In order to avoid redundancy and signal saturation caused by multiple substitutions at the same site, only Ks values within the range of 0.05–5 were considered, respectively. Gaussian mixture models were then fitted to the resulting frequency distributions by means of two alternative methods. First, the densityMclust function from the R mclust v5.3 package^79^. The Bayesian information criterion was used to determine the best-fitting model for the data, including the optimal number of Gaussian components up to a maximum of nine. Alternatively, Gaussian mixture models were fitted to the Ks values by means of the normalmixEM() function from the R mixtools v1.2 package, following a similar procedure to that described in Li et al.^80^. First, we determined for each Ks distribution the number of Gaussian components (k) using the boot.comp() function, which performs parametric bootstrap for sequentially testing the number of components in various Mixture Models. Specifically, we performed parametric bootstraps with 1000 replicates of the likelihood ratio statistic for testing the null hypothesis of a k-component fit versus the alternative hypothesis of a (k + 1)-component fit, using a significance level of 0.01. For each distribution, we tested the presence of one to nine components. The number of components determined in this first step was used to fit a mixture of Gaussian models to the Ks distribution, using the normalmixEM() function with the following parameters: maxit = 1e-30, maxrestarts = 1e−3, epsilon = 1e−10. Each retrieved peak potentially corresponding to an episodic event of genome duplication is characterized by an age expressed in Ks values equals to the mean of the Gaussian mixture component.

### Sequence and phylogenetic analysis of the *RBCL* gene family

Multiple aminoacidic sequence alignments were obtained using MUSCLE^77^. Multiple codon sequence alignments were obtained using PRANK v.140603 with the settings -codon and -F^72,73^. ML phylogenetic analyses were performed on the basis of codon alignments. Prior to the analysis, the best fit nucleotide substitution model was inferred using JModelTest v2.1^81^. The General Time reversible (GTR)^82^, + G, modelling heterogeneity in nucleotide substitution rates across positions in the alignment by means of a Gamma distribution with eight categories and an alpha shape parameter of 2 was selected as the best fit model by the AIC test, and used to reconstruct ML trees by means of the PhyMLv3.1 software^76^. To optimize the search of the most likely tree, the best of NNI & SPR (NNI, nearest-neighbor interchange; SPR, subtree pruning and regrafting) was selected^76^. Statistical significance on the retrieved topology was assessed by means of the Shimodaira–Hasegawa-like approximate likelihood ratio test^83^.

## Supplementary information

Supplementary information.

Supplementary figures.

Supplementary tables.
