# Supplementary material for: Evolutionary analysis of the Moringa oleifera genome reveals a recent burst of plastid to nucleus gene duplications
Source: Sci Rep. 2020 Oct 19;10:17646. doi: 10.1038/s41598-020-73937-w (PMC7573628; doi:10.1038/s41598-020-73937-w)
Supplement: Supplementary file 1 — Supplementary information. [file 41598_2020_73937_MOESM1_ESM.docx]

**Title Page**

**Evolutionary analysis of the *Moringa oleifera* genome reveals a recent burst of plastid to nucleus gene duplications**

**Authors:** José Ojeda López**^†^**, Juan Pablo Marczuk-Rojas**^†^**, Oliver Aleksandrei Polushkina, Darius Purucker, María Salinas Navarro, Lorenzo Carretero-Paulet*

**Address**: Department of Biology and Geology. University of Almería. Ctra. Sacramento s/n

04120 Almería. Spain.

* Corresponding author: [lpaulet@ual.es](mailto:lpaulet@ual.es) (ORCID Number: 0000-0001-6697-827X).

**^†^** These authors have contributed equally to this work and share first authorship.

**Legends for Supplementary material**

**Supplementary Figure S1: Histogram of the distribution of plastid DNA insertion sizes in the Moringa genome.** Bin width is set to 250. The location of the mean and the median are indicated with blue and red vertical dashed lines, respectively.

**Supplementary Table S1: Summary of plant genome versions used in this study.**

**Supplementary Table S2. Results of fitting the Badirate GD models of gene family turnover to an orthogroup classification in Moringa and 10 other flowering plant species.** Families for which the Moringa-specific branch model fitted significantly better than the second-best model according to the AIC test > 2.7, with a net gene gain positive, or negative, corresponding to expanded and contracted gene families are shown in light green and yellow colors, respectively.

**Supplementary Table S3. GO functional categorization of genes belonging to orthogroups identified in the BadiRate analyses as significantly contracted in Moringa.** For each generic GO term, the table shows the number of counts among genes belonging to Moringa-specific contracted genes families, the total number of counts in the genome, the Fisher’s exact test of differential distribution (raw and adjusted p-values, both by Benjamini-Hochberg or Bonferrroni), as well as the direction of the differential representation (over- or under-represented). At the end of the table showing the Fisher’s tests, Moringa expanded orthogroups are listed with their individual gene members and associated GO and KEGG annotations.

**Supplementary Table S4. GO functional categorization of genes belonging to orthogroups identified in the BadiRate analyses as significantly expanded in Moringa.** For each GO term, the table shows the number of counts among genes belonging to Moringa-specific expanded genes families, the total number of counts in the genome, the Fisher’s exact test of differential distribution (raw and adjusted p-values, both by Benjamini-Hochberg or Bonferrroni), as well as the direction of the differential representation (over- or under-represented). At the end of the table showing the Fisher’s tests, Moringa expanded orthogroups are listed with their individual gene members and associated GO and KEGG annotations.
