# Supplementary figures and images for: Evolutionary analysis of the Moringa oleifera genome reveals a recent burst of plastid to nucleus gene duplications
Source: Sci Rep. 2020 Oct 19;10:17646. doi: 10.1038/s41598-020-73937-w (PMC7573628; doi:10.1038/s41598-020-73937-w)

## Slide 1
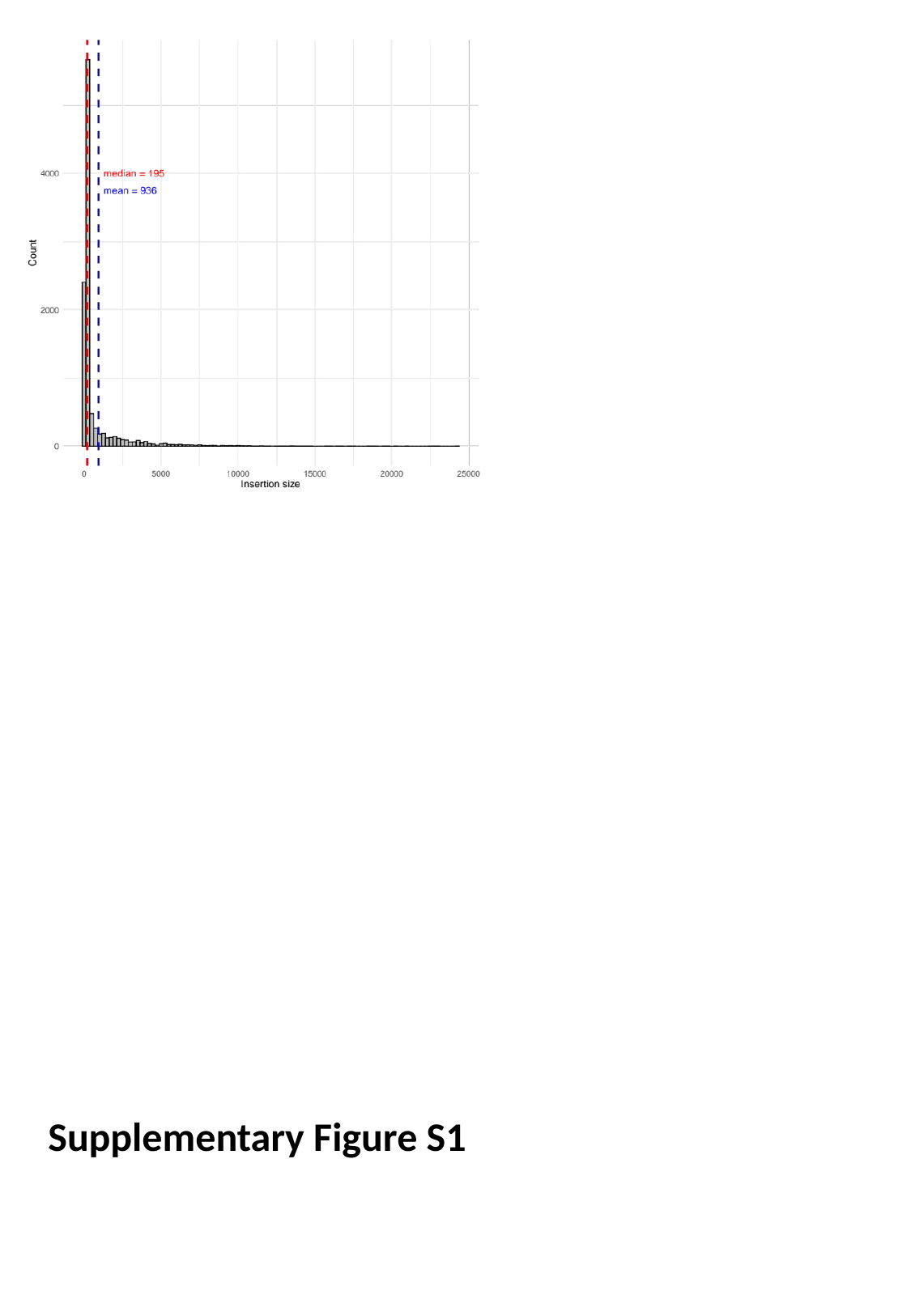

Supplementary Figure S1

Supplement: Supplementary file 2 — Supplementary figures. [file 41598_2020_73937_MOESM2_ESM.pptx]
